# Knowledge concept recognition in the learning brain via fMRI classification

**DOI:** 10.3389/fnins.2025.1499629

**Published:** 2025-03-21

**Authors:** Wenxin Zhang, Yiping Zhang, Liqian Sun, Yupei Zhang, Xuequn Shang

**Affiliations:** ^1^School of Computer Science, Northwestern Polytechnical University, Xi'an, China; ^2^Big Data Storage and Management MIIT Lab, Xi'an, China

**Keywords:** knowledge concept recognition, deep learning, fMRI classification, brain identification, learning science

## Abstract

Knowledge concept recognition (KCR) aims to identify the concepts learned in the brain, which has been a longstanding area of interest for learning science and education. While many studies have investigated object recognition using brain fMRIs, there are limited research on identifying specific knowledge points within the classroom. In this paper, we propose to recognize the knowledge concepts in computer science by classifying the brain fMRIs taken when students are learning the concepts. More specifically, this study made attempts on two representation strategies, i.e., voxel and time difference. Based on the representations, we evaluated traditional classifiers and the combination of CNN and LSTM for KCR. Experiments are conducted on a public dataset collected from 25 students and teachers in a computer science course. The evaluations of classifying fMRI segments show that the used classifiers all can attain a good performance when using the time-difference representation, where the CNN-LSTM model reaches the highest accuracy. This research contributes to the understanding of human learning and supports the development of personalized learning.

## 1 Introduction

The recognition of knowledge concepts in the brain aims to identify the contexts that are learning or thinking, which is critical for human learning understanding (Seguin et al., 2023). It is useful in many current research fields, including the neural pattern in education (Meshulam et al., 2021), intelligent human-machine interface (Allen et al., 2022), and brain disorder treatment (Chianese et al., 2021). Hence, knowledge concept recognition (KCR) has become an emerging direction in recent years due to the quick development of brain science and its applications.

In general, KCR involves scanning the active brain to acquire imaging data during different task states and using classification techniques to identify the corresponding brain images for various task states (Zhang et al., 2023). The techniques of brain imaging acquisition can be invasive or non-invasive, such as functional magnetic resonance imaging (fMRI) and positron emission tomography (PET) (Chang et al., 2022). Wherein, fMRI is widely used in investigations of brain functions due to its high spatial resolution and non-invasive acquisition. The fMRI images are often acquired by brain scanning for many timestamps, where one picture is obtained at each timestamp (Allen et al., 2022; Meshulam et al., 2021). Hence, fMRI data usually involves a sequence of images, where each pixel in the image measures the Blood Oxygenation Level Dependent (BOLD) signal at a brain location. With the time-series fMRI, we acquire the brain activity using the changes of BOLD values in the brain (Allen et al., 2022).

With the assumption of different BOLD patterns in fMRI for different cognitive concepts, the KCR focused is usually formulated into fMRI classification (Feng et al., 2022). In recent years, many approaches have been developed to identify fMRI images. Zhang et al. (2022b) proposed a multi-instance model with contrastive learning to identify non-math students and Alzheimer's disease. Qiang et al. (2023) knitted the VAE-GAN method by integrating variational auto-encoder(VAE) and generative adversarial net(GAN) to implement fMRI augmentation for Attention Deficit Hyperactivity Disorder(ADHD) classification. Zhang et al. (2022a) used a novel feature selection method by the difference between step distribution curves and utilized a multilayer perception pre-trained by a VAE for identifying the Autism spectrum disorder (ASD). However, the current studies of fMRI classification rarely consider the problems in the classroom (Mason and Just, 2016). Li et al. (2023) used spatio-temproal graph neural networks to identify the learning disability from brain graphs, while the identification of concepts learned is few touched (Lei et al., 2023; Mason and Just, 2016). Wang et al. (2013) developed a multi-voxel fMRI pattern analysis to identify the difference between abstract and concrete concepts by using a logical regression classifier, where the fMRIs are yielded by asking different words. Mason and Just (2016) used the naive Bayes classifiers to identify the physics concepts from fMRI, showing the discriminability of the brain activation signature.

**Figure 1:**
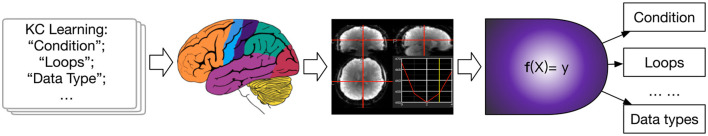
The framework of our study. From courses, the brain receives stimulation of concepts, resulting in fMRIs. This study explore creating the mapping *f* to identify what concept is learning in the brain.

The recent study shows neural representation could predict learning outcomes in students taking a computer science (CS) course (Meshulam et al., 2021). To explore the process, in this paper, we use machine-learning-based fMRI classification methods for KCR in the CS classroom. Our KCR tasks are focused on identifying those concepts learned in student learning, where the concepts involve basic knowledge points of programming (Meshulam et al., 2021). Our study framework is shown in Figure 1. The contributions are 3-folds:

(1) This paper contributes to the topic of KCR from fMRI in education, which aims to discover the cognitive pattern in the learning brain. It can help trace the knowledge in the brain (Zhang et al., 2020) and make a personalized learning plan.

(2) Two strategies of fMRI classification are discussed, including voxel-based and temporal difference-based methods. For the two methods, traditional machine learning models and deep neural networks are evaluated, respectively.

(3) The CNN-LSTM model integrating convolutional neural networks (CNN) and long short-term memory (LSTM) are utilized to extract the spatial and temporal features from the BOLD variances, resulting in a better performance than other methods.

The remainder of this paper is organized as follows. Section 2 investigates related works of the KCR and fMRI classification. The used dataset is introduced in Section 3. The traditional classifiers and CNN-LSTM are introduced in Sections 4, where both voxel-based and difference-based representation are also given, respectively. Experimental results are presented and analyzed in Section 5. Finally, Section 6 concludes this study.

## 2 Related work

### 2.1 Concept recognition

Recognition of knowledge concepts in brain imaging data involves scanning the brain under different learning task states to obtain brain imaging data. Then, utilizing classification techniques to identify the corresponding brain images for different task states. This is crucial for understanding human learning (Bréchet et al., 2019) and represents a new direction emerging in recent years. According to different types of brain imaging data, KCR can be implemented via different techniques, including functional magnetic resonance imaging (fMRI), structural magnetic resonance imaging (sMRI), and electroencephalography (EEG). In recent years, with the development of fMRI and sMRI (Khvostikov et al., 2018) technologies, researchers have been able to obtain detailed information about brain function and structure, leading to significant advances in the field of cognitive neuroscience. Zeithamova et al. (2019) proposed a geometric deep learning framework for cross-modal brain anatomy and functional mapping, which is important for understanding the relationship between brain structure and function, as well as studying neurological disorders. Additionally, in the identification of EEG images, Li et al. (2016) encapsulated multi-channel neurophysiological signals into grid-like frames through wavelet transform and spectrogram transform. They further designed a hybrid deep learning model, combining Convolutional Neural Networks (CNN) and Recurrent Neural Networks (RNN), to extract task-related features, explore inter-channel correlations, and incorporate contextual information from these frames. However, there is few studies of recognizing the KC from fMRIs in a classroom (Zhang et al., 2022c). In general, KCR can be cast as a fMRI classification problem simply (Zhang et al., 2023).

### 2.2 FMRI classification

Currently, there are many pathological analyses (Wang et al., 2021), neuro-disease diagnoses (Ronicko et al., 2020), and pattern recognition (Wang et al., 2019) methods based on fMRI data. They are mainly divided into traditional machine learning-based methods and deep learning-based methods, where deep learning methods can be further categorized into voxel-based classification methods based on convolutional neural networks and graph-based representation classification methods based on functional connectivity.

Traditional machine learning methods were initially applied to the correlation analysis of brain regions and cognitive functions in fMRI data. By examining the response of each voxel in the fMRI data of subjects under different stimuli (whether the voxel is “activated,” measured by the change in neural metrics at that point), mapping the voxels to cognitive stimuli was attempted. However, this approach ignored the correlation between voxels in different locations. Multi-voxel pattern analysis (MVPA) (Weaverdyck et al., 2020) applies multivariate analysis to multiple voxels in fMRI data to improve the representation of voxel relationships. Therein, linear discriminant analysis and support vector machines are also used in the comparisons. Kuncheva et al. (2010) proposed a support vector machine model based on random subspaces and compared its performance with other machine learning classifiers for fMRI classification. Ryali et al. (2010) achieved the dual objectives of discriminating brain regions and classifying fMRI data using logistic regression combined with L1 and L2 regularization and other machine learning techniques. However, the traditional machine learning-based fMRI classification is insufficient in representation learning.

Deep learning algorithms, benefiting from the power of neural networks, show the better performance in the fMRI classification. On the one hand, voxel-based deep learning methods have achieved research results in various fields. Feng et al. (2022) proposed a method that combines Deep Feature Selection (DFS) and Graph Convolutional Networks (GCN), to classify ASD and developing control groups, significantly improving the prediction performance. Researchers then focused on the temporal nature of fMRI data, integrating sequence learning ideas such as LSTM and Markov processes into fMRI data classification. These deep learning methods have shown improvement compared to traditional machine learning methods, but generally ignored the structure information, such as the interregional correlations of the brain (Li et al., 2023). On the other hand, functional connectivity (FC) is achieved based on the voxel-wise time series of fMRI images, reflecting the functional spatio-temporal relationships between brain regions (Lurie et al., 2020). Dynamic connectivity analyses (Zarghami and Friston, 2020) are investigated the neuronal basis of metastability. Generally, two main methods are often used to calculate FC: correlation analysis (Liégeois et al., 2020) and clustering decomposition (Cribben and Yu, 2017). In the former, brain regions with strong correlations are generally considered to be functionally connected, such as Pearson product-moment correlation and Spearman's rank correlation (Lei et al., 2023). The latter clusters brain regions to be functionally connected. FC has been utilized for diagnosing Autism Spectrum Disorder (ASD) (Shao et al., 2021), Alzheimer's disease (AD) (Zuo et al., 2024b, 2023a), and cognitive impairment (Zuo et al., 2024a, 2023b).

Besides, graph-based learning methods have been developed to identify the brain status, since the brain graph can be conducted by functional connectivity matrix (Bessadok et al., 2022; Zong et al., 2024). Kim and Ye (2020) develop an approach for graph analysis based on resting-state fMRI to diagnose the spectrum disorders. To consider the sequential features, Lei et al. (2023) developed a Spatio-Temporal Graph Convolutional Network (ST-GCN) for brain representation.

However, there is few studies that are to diagnose whether a KC from a computer course has been mastered by student (Zhang et al., 2022c). Inspired by this, this study aims to learn and encode both temporal and spatial information into the graph structure for subsequent spectral graph convolution methods to learn concepts from the graph structure. With the different representation, the study is to recognize the KC from brain fMRI, exploring a novel possibility of knowledge diagnoses for education (Zhang et al., 2020).

## 3 Problem definition and the used public dataset

To be more clear, we definite the problem of KCR here. Let *X* be a input fMRIs and *y* be the knowledge concept (KC) learned in the brain. The KCR problem finds a function *f* to :


(1) $$\begin{array}{l} minimize\|f\left(X\right)-y{\|}_{2}^{2} \end{array}$$

such that *f* can identify the KC in the brain. The studies on data analysis has been develop in the work of Meshulam et al. (2021). But they did not develop the KCR in their works while focusing on analyzing the relationship between students and experts.

**Figure 2:**
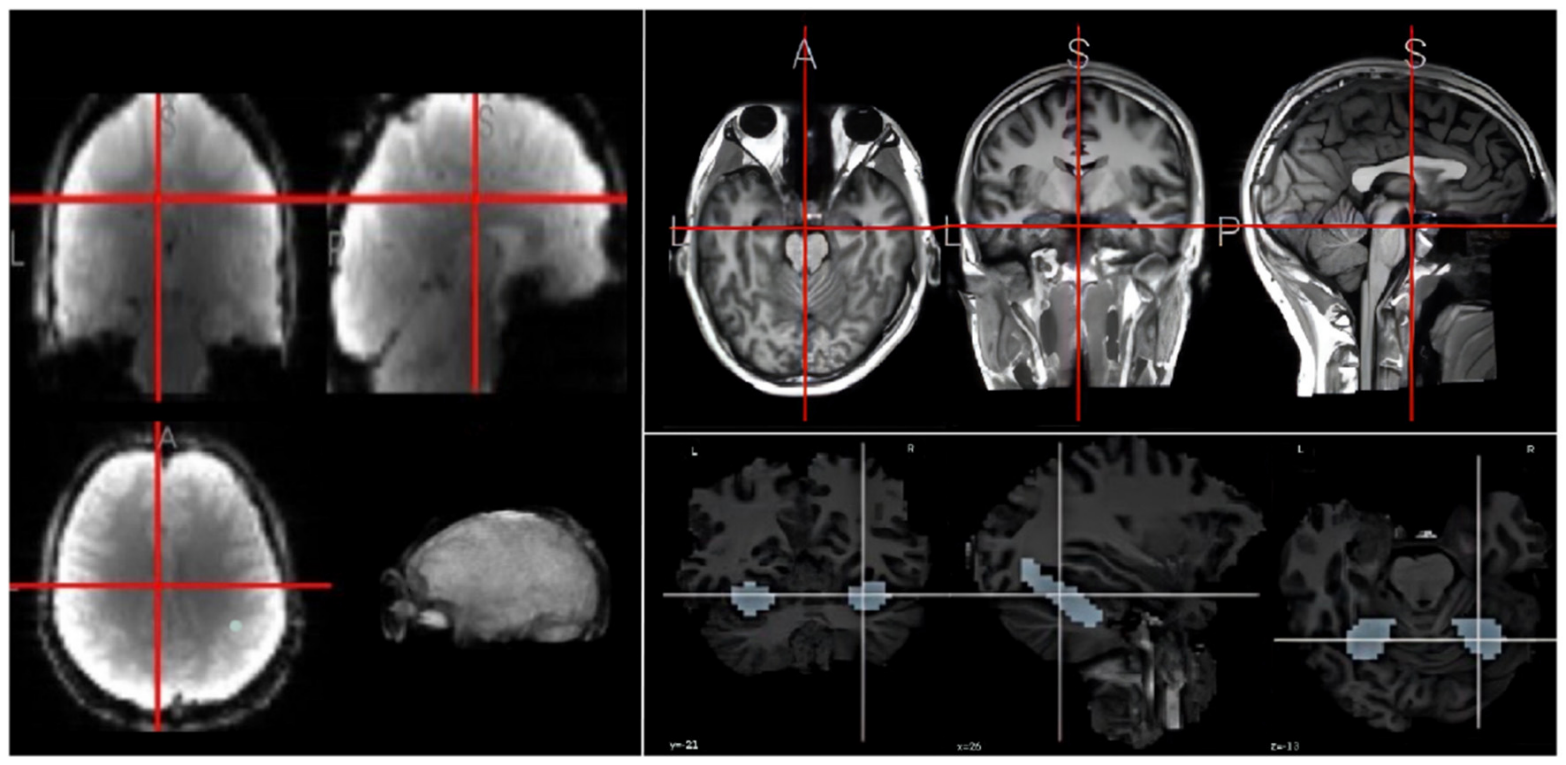
An example fMRI **(left)**, MRI **(right upper)**, and hippocampus **(right down)**.

Inspired by the research work of Meshulam et al. (2021), this study uses their published datasets collected brain images from 25 participants,^1^ as shown in Figure 2. The participants in this study consisted of 20 students and 5 teachers. The short description is as follows: The students underwent six scans, while the teachers participated in a single fMRI scan. During the first five scans, students viewed lectures from the course “An Introduction to Computer Science,” which covered topics such as conditions and loops, libraries and functions, abstract data types, performance, and the theory of computing. In the sixth scan, both students and teachers watched a knowledge review video summarizing the material from the previous weeks and then took an exam. NOTE THAT there are many limitations in data collection, such as student's requirements and the number of students in the classroom, leading to the small size of dataset. More details about this dataset is refereed to the paper published by Meshulam et al. (2021).

Due to the complexity, this study focuses on the hippocampus. Learning in the brain is a complex system, which is affected by many brain regions, such as the hippocampus, the prefrontal cortex, and the parietal lobe (Gavazzi et al., 2023). However, this study aims to recognize the abstract concepts learned in the brain. Since the hippocampus is one of the most important brain regions in concept abstract (Courellis et al., 2024), we here focused on hippocampus for learning KC from videos and extracted the corresponding fMRI patches. For the convenience in model training, we extracted 668 fMRI voxels for the hippocampus. To mitigate the small sample issue, we divided the fMRI sequence into short fragment along time with about 15 time stamps per segment. The category labels correspond to five computer-science concepts.

## 4 The used fMRI classification methods

### 4.1 Traditional voxel-based machine learning methods

#### 4.1.1 Data pre-processing

The fMRI images were here pre-processed as follows. First, motion correction was applied using MCFLIRT (Jenkinson et al., 2002) to address rearrangements between images, correcting for motion both within and across questions. Next, a joint registration of functional and anatomical images was performed for each participant using a 12-degree linear transformation. The anatomical images were then normalized to the standard brain template defined by the Montreal Neurological Institute's 152-brain average, followed by a 6-degree-of-freedom non-linear registration from structural to standard space. Finally, slice timing correction was conducted.

Given the complex spatial structure of the hippocampus examined in this study, the 668 voxels were flattened into a one-dimensional format. The entire dataset was divided into different time steps, usually with a time step of 15. This process generated the preliminary data required for the model, where each sample is represented as a tensor of size 668 × 15, where the 668 voxels represent the hippocampus and 15 represents the data fragment having 15 time steps. To mitigate the dimensional impact between metrics and enhance the comparability of data indicators, data standardization is essential. This paper employs the following normalization formula:


(2) $$\begin{array}{l} x^{\prime }=\frac{x-\mu }{\sigma } \end{array}$$

where *x* indicates the raw data, μ and σ are the mean and standard deviation of *x*, and *x*′ is the normalized data. In addition, we used two normalizations for individual samples and the entire category.

#### 4.1.2 Traditional machine learning models

The traditional classification models utilized in this research are the Support Vector Machine (SVM) and the k-Nearest Neighbors (KNN) algorithm (Bhutta et al., 2023). Both methods are often employed for classification (Zhang et al., 2021). The parameters used in experiments are introduced in the specific evaluations.

### 4.2 BOLD differences-based spatio-temporal deep neural networks

#### 4.2.1 Data processing for difference computation

In the preceding experiments, the flattening of all voxels into a one-dimensional format resulted in the loss of their overall spatial characteristics. To account for spatial features, this study employed a two-dimensional representation of fMRI images. To account for temporal features, this paper implements differential operations on the original data within the temporal dimension, expressed mathematically by *X*_diff_ = *X*_*t*_ − *X*_*t*−1_ where *X*_*t*_ is the *t*-slice in a fMRI. Then, normalization is performed on the time-difference.

#### 4.2.2 The CNN-LSTM model

**Figure 3:**
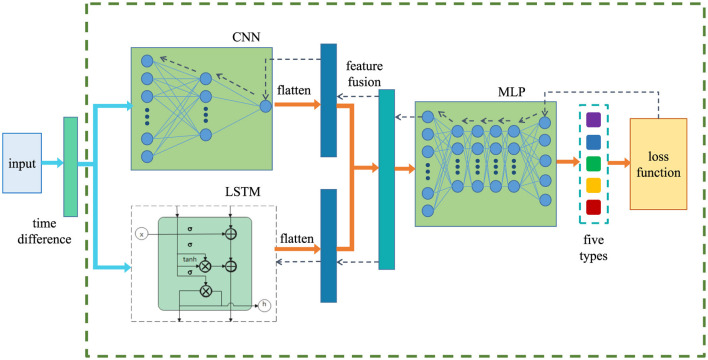
The used CNN-LSTM framework, where the input size is depended on the classification tasks in hand, while the loss function is the cross entropy (Mao et al., 2023). The dashed arrows in the figure indicate the direction of back-propagating.

To extract robust spatio-temporal features, we develop a spatio-temporal network model, CNN-LSTM, as illustrated in Figure 3. This model comprises five distinct components: data enhancement, convolutional neural network (CNN), long short-term memory neural network (LSTM), feature fusion, and fully connected neural network. In CNN-LSTM, Convolutional neural networks (CNN) can extract spatial features of the model; long short-term memory neural networks (LSTM) can extract the temporal sequence features of the data; combining CNN and LSTM together can achieve the extraction of model spatio-temporal features.

Given a set of fMRI data *X* = {*X*_1_, *X*_2_, *X*_3_, ..., *X*_*n*_}, each *X*_*i*_ = [*X*_*i,j*_] represents the BOLD value of the *j*-th voxel in the hippocampus at the time step *t*_*i*_. The model we proposed tries to learn an optimal model *F* so that *F*(*X*_*i*_) is directly mapped to its corresponding label *y*_*i*_. There are five concept types of *y*_*i*_, which correspond to the fMRI images under specific computer course tasks. The CNN-LSTM model described in this paper can be outlined through the following steps:

1. Process the fMRI images using a time difference operation to obtain the data *X*.
2. Apply a convolution to the enhanced X through the CNN layer to yield *X*^*C*^.
3. Pass the enhanced *X* through the LSTM layer with *k* modules to obtain *X*^*L*^.
4. Fuse *X*^*C*^ and *X*^*L*^ to obtain *X*^*M*^.
5. Feed the obtained *X*^*M*^ into a fully connected neural network (FCN) for classification.

In summary, the prediction results of this framework are achieved by


(3) $$\begin{array}{l} y_{i}=MLP\left(CNN\left(X_{i,j}\right)+LSTM\left(X_{i,j}\right)\right) \end{array}$$

where *y*_*i*_ is the label; MLP indicates the Multi-Layer Perceptron, while LSTM is Long Short-Term Memory. Note that LSTM is a traditional approach to handle the time sequence data, while the Transformer has been well known for their strong capabilities in parallel computation, global context modeling, and adaptability to sequence data (Han et al., 2021). However, the fMRI datasets used here is hard to train Transformer due to its complexity. Hence, in this study, we just explored the KCR by using LSTM (Zhang et al., 2022c).

## 5 Experiment results

### 5.1 Evaluation metrics

We evaluated the used classification methods for KCR by common metrics. For this multi-label classification, we calculated the metric by considering the samples belonging to the target class as positive samples. Let *TP*, *TN*, *FP*, and *FN* be true positive, true negative, false positive, and false negative for classification result, respectively. The following metrics evaluate model performance on the test set:


$$\begin{array}{l} \begin{array}{c} {\text{Precision}}_{i}=\frac{TP_{i}}{TP_{i}+FP_{i}}\\ {\text{Recall}}_{i}=\frac{TP_{i}}{TP_{i}+FN_{i}}\\ {\text{F\_1 Score}}_{i}=2\cdot \frac{Precision_{i}\cdot Recall_{i}}{Precision+Recall}\\ \text{Accuracy}=\frac{TP+TN}{TP+TN+FP+FN}\\ \text{Cohen's Kappa Coefficient}=\frac{P_{o}-P_{e}}{1-P_{e}} \end{array} \end{array}$$

where *i* indicates the *i*-th class, *P*_*o*_ is the observed agreement, and *P*_*e*_ is the expected agreement by chance. The 10-fold cross-validation is adopted to achieve the classification accuracy. The process is as follows: we randomly divided the datasets into 10 folds where 9 folds are for training classifiers and the rest fold is for computing test accuracy. Finally, we reported the average values from the 10 folds. To evaluate the data imbalance issue, we also computed the Micro-average and Macro-average computed as in the previous work (Zhang et al., 2022d). In this study, we tried to ensure that the number of samples across classes was approximately balanced, leaving the issue of data imbalance for future consideration.

### 5.2 Voxel-based classification evaluation

#### 5.2.1 Evaluations on traditional classifiers

We classified fMRI data under five concept categories using traditional classification models, i.e., SVM and KNN. The classification results are shown in Table 1, together with their parameter settings. From the results, it can be seen that the SVM model performs well, achieving an accuracy of 78%. While the performance of KNN is relatively poor, with only 42% accuracy. This might be because the KNN model is relatively simple and considers only the “distance” factor, leading to lower accuracy.

**Table 1:** Results of using SVM and KNN algorithms with different normalizations, where the empirical parameters are set for high prediction accuracy.

**Classifier**	**Parameter setting**	**Accuracy**	
		**Region normalization**	**Category normalization**
SVM	decision_function_shape = “ovr,” kernel = “linear,” C = 10, max_iter = 1,000	0.76	0.78
KNN	n_neighbors = 30, weights = “uniform,” p = 2	0.39	0.42

**Figure 4:**
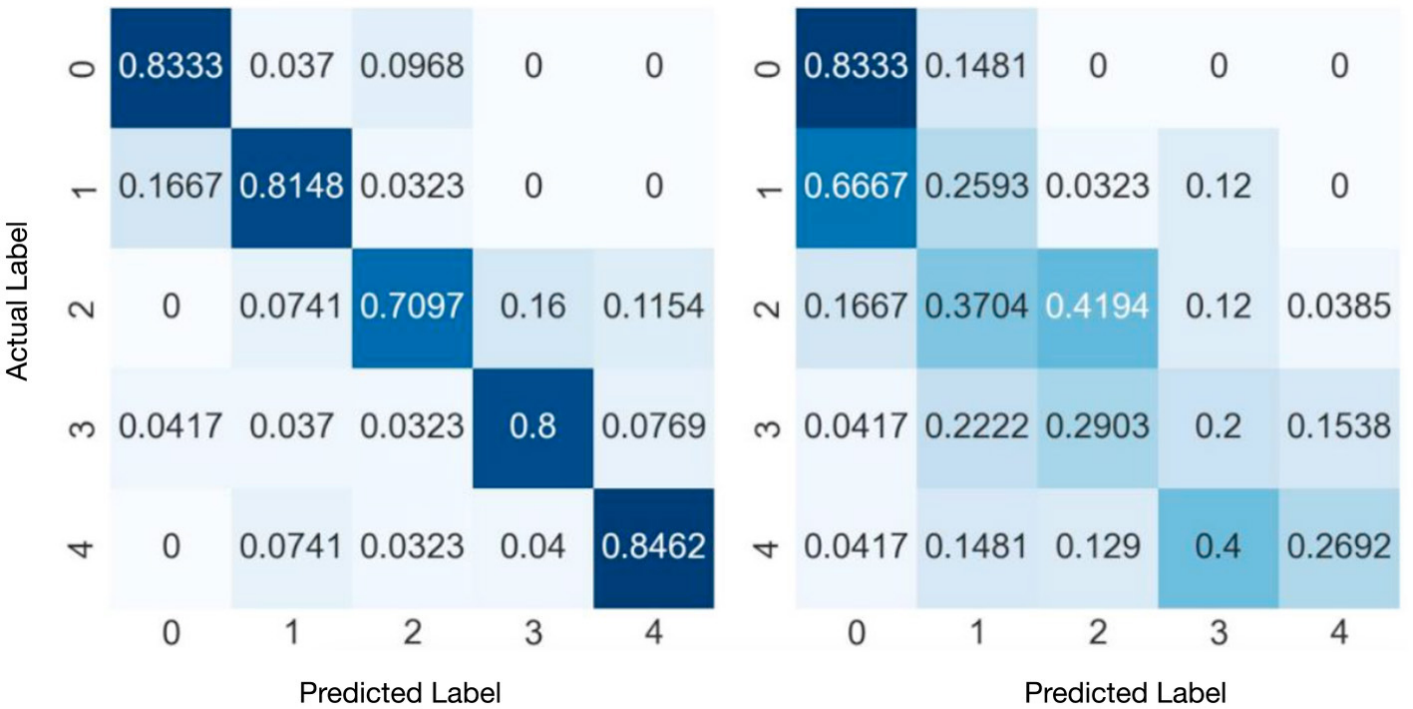
Confusion matrixes of SVM **(right)** and KNN **(left)** with category normalization. The vertical axis is the actual labels, while the horizontal axis is the predicted labels.

Figure 4 presents the confusion matrices of SVM and KNN obtained from the experiments. The results indicate that the SVM achieves relatively high classification accuracy across all classes. On Category “2,” i.e., abstract data types, SVM has relatively lower performance. The classification accuracy for the remaining categories exceeds 80%. While, KNN's overall performance is relatively poor from the left matrix. Category “1” is significantly misclassified into Category “2.”

Table 2 presents the experimental results of the investigation of the impact of time step {1, 3, 5}. It is observed that reducing the time step can result in an increase in training accuracy for KNN and SVM. That is due to the increasing number of training samples.

**Table 2:** Comparison of SVM and KNN with different time steps, where the empirical parameters are set to obtain higher classification accuracy.

**Classifier**	**Parameter setting**	**Category normalization**		
		**Time step**		
		**5**	**3**	**1**
SVM	decision_function_shape = “ovr,” kernel = “linear,” C = 10, max_iter = 1,000	0.65	0.68	0.69
KNN	n_neighbors = 30, weights = “uniform,” p = 2	0.53	0.61	0.71

#### 5.2.2 Evaluations on deep learning models

We conducted experiments by using MLP and (CNN1D+LSTM) × MLP to show the prediction performance. The results are shown in Table 3 with their using parameters. As is shown, we could achieve the following observations and conclusions. (1) MLP consisting of a 5-layer neural network was trained to directly classify the flattened data vector, achieving an accuracy of 74%. (2) Considering the temporal features, we evaluated the model (CNN1D+LSTM) × MLP which combines CNN1D and LSTM on flattened vectors and then connect to MLP, achieving 81%.

**Table 3:** Parameters and comparison of MLP and (CNN+LSTM) × MLP, where the empirical parameters are set to obatin higher classification accuracy.

**Classifier**	**Parameter setting**	**Accuracy**
MLP	(10,020,512,256,128,64,5), optimizer = “adam,” Epoch = 3,000	0.74 ± 0.023
(CNN1D+LSTM) × MLP	Conv1d(in_channels = 668, out_channels = 100, kernel_size = 1) LSTM(input_size = 668, hidden_size = 100, num_layers = 5, bias = True, batch_first = True, dropout = 0.25, bidirectional = False) (3,000,512,256,64,5), Dropout = 0.2, Epoch = 3,000	0.81 ± 0.031

**Figure 5:**
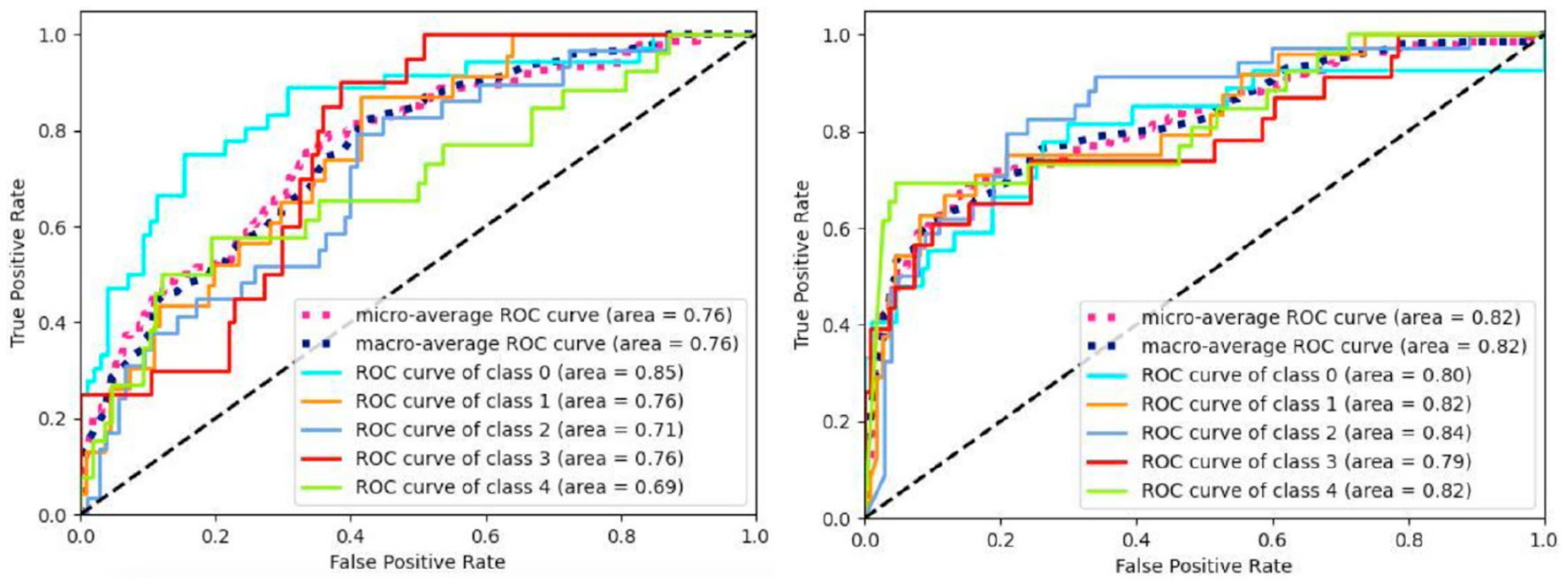
ROC curves of MLP **(left)** and (CNN+LSTM) × MLP **(right)**.

Figure 5 displays the ROC curves obtained from the experiment. The results manifest that (CNN1D+LSTM) × MLP reaches the better performance than MLP in terms of ROC and AUC. Besides, we aggregate the per-class metrics into the micro/macro-average ROC curve and AUC. Their results show that (CNN1D+LSTM) × MLP is better than MLP. All results imply the effectiveness of integrating spatial and temporal features.

### 5.3 Time-difference based classification evaluation

This subsection evaluates the time difference based methods. To check effectiveness of each component, we employed four different models for comparisons, shown in Table 4, together with their used parameters. Note that we adjusted the model in the training process to seek their best performance in our experiments.

**Table 4:** Parameters and accuracy of the combinations of MLP, CNN, LSTM, and LSTM.

**Classifier**	**Parameter setting**	**Accuracy**
MLP	(10,020,512,256,128,64,5), optimizer = “adam,” Epoch = 3,000	0.88 ± 0.016
CNN+MLP	Conv[1(3,3), strides=1, padding = “same,” use_bias = False] Conv2D[1,(3,3), strides = 1, padding = “same,” use_bias = False] (20,040,512,256,64,5), Dropout = 0.2, Epoch = 500	0.91 ± 0.024
LSTM+MLP	LSTM(input_size = 668, hidden_size = 100, num_layers = 5, bias = True, batch_first = True, dropout = 0.25, bidirectional = False) (11,520,512,256,64,5) Dropout = 0.2, Epoch = 500	0.92 ± 0.021
(CNN+LSTM) × MLP	Conv(in_channels = 668, out_channels = 100, kernel_size = 1) LSTM(input_size = 668, hidden_size = 100, num_layers = 5, bias = True, batch_first = True, dropout = 0.25, bidirectional = False) (3,000,512,256,64,5), Dropout = 0.2, Epoch = 500	0.94 ± 0.029

The standard deviation are obtained by 10 folds.

From Table 4, these observations reveal that both CNN+MLP and LSTM+MLP reach better performance than MLP, while (CNN+LSTM) × MLP achieves the best performance. Besides, the MLP model attained good results after 3,000 training iterations, while other models achieved comparable performance after only 500 training iterations. This implies that more powerful feature representations not only enhance the classification accuracy of the models but also accelerate their convergence. However, the less steps likely incurs higher standard deviations.

In Table 5, we computed the performance evaluation results of each model utilizing time difference in terms of precision, accuracy, recall, F1 score, and Cohen's Kappa coefficient. The results indicate that considering either spatial or temporal features enhances classification performance. Moreover, the integration of both types of features appears to yield superior results compared to the use of either feature type alone. That implies that learning concept in the brain is not only a structural activity but also a temporal activity.

**Table 5:** Evaluation metrics of compared methods, where the accuracy is from Table 4.

**Classifier**	**Precision**	**Accuracy**	**Recall**	**F1 Score**	**Cohen's Kappa coefficient**
MLP	0.90	0.88	0.84	0.867	0.67
CNN+MLP	0.89	0.91	0.86	0.875	0.76
LSTM+MLP	0.91	0.92	0.90	0.905	0.74
(CNN+LSTM) × MLP	**0.93**	**0.94**	**0.91**	**0.935**	**0.81**

## 6 Conclusion

In this study, we proposed utilizing learning methods for knowledge concept recognition (KCR), a compelling problem in brain decoding. We implemented two approaches to data preprocessing: raw voxel sequences and time-difference sequences. When utilizing time-difference sequences, the results show significantly improved performance, compared to using the voxel sequences. Experimental results show the consideration of both spatial and temporal features proves to be particularly effective in fMRI classification for KCR.

In future work, we will consider a bigger fMRI datasets for learning science and use more explainable feature extract model and deep models (Ning et al., 2023). To address the small data-size problem, we will adopt the federated learning framework to have fMRI analyses with many other institute (Zhang et al., 2025, 2024). Finally, toward a personalized learning plan, the variability between students will be worthy to consider in the future.

## Data Availability

The original contributions presented in the study are included in the article/supplementary material, further inquiries can be directed to the corresponding author.

## Footnotes

^1^ https://openneuro.org/datasets/ds003233/versions/1.2.0
